# Fluid and Tissue Biomarkers of Lewy Body Dementia: Report of an LBDA Symposium

**DOI:** 10.3389/fneur.2021.805135

**Published:** 2022-01-31

**Authors:** Gregory D. Scott, Moriah R. Arnold, Thomas G. Beach, Christopher H. Gibbons, Anumantha G. Kanthasamy, Russell M. Lebovitz, Afina W. Lemstra, Leslie M. Shaw, Charlotte E. Teunissen, Henrik Zetterberg, Angela S. Taylor, Todd C. Graham, Bradley F. Boeve, Stephen N. Gomperts, Neill R. Graff-Radford, Charbel Moussa, Kathleen L. Poston, Liana S. Rosenthal, Marwan N. Sabbagh, Ryan R. Walsh, Miriam T. Weber, Melissa J. Armstrong, Jee A. Bang, Andrea C. Bozoki, Kimiko Domoto-Reilly, John E. Duda, Jori E. Fleisher, Douglas R. Galasko, James E. Galvin, Jennifer G. Goldman, Samantha K. Holden, Lawrence S. Honig, Daniel E. Huddleston, James B. Leverenz, Irene Litvan, Carol A. Manning, Karen S. Marder, Alexander Y. Pantelyat, Victoria S. Pelak, Douglas W. Scharre, Sharon J. Sha, Holly A. Shill, Zoltan Mari, Joseph F. Quinn, David J. Irwin

**Affiliations:** ^1^Department of Pathology, Oregon Health and Science University, Portland, OR, United States; ^2^Department of Pathology and Laboratory Services, VA Portland Medical Center, Portland, OR, United States; ^3^Graduate Program in Biomedical Sciences, School of Medicine M.D./Ph.D. Program, Oregon Health and Science University, Portland, OR, United States; ^4^Civin Laboratory for Neuropathology and Brain and Body Donation Program, Banner Sun Health Research Institute, Sun City, AZ, United States; ^5^Department of Neurology, Beth Israel Deaconess Medical Center and Harvard Medical School, Boston, MA, United States; ^6^Department of Physiology and Pharmacology, Center for Brain Sciences and Neurodegenerative Diseases, University of Georgia, Athens, GA, United States; ^7^Amprion Inc., San Francisco, CA, United States; ^8^Department of Neurology, Amsterdam University Medical Center (UMC), Alzheimer Center Amsterdam, Vrije Universiteit Amsterdam, Amsterdam, Netherlands; ^9^Department of Pathology and Laboratory Medicine, Perelman School of Medicine, University of Pennsylvania, Philadelphia, PA, United States; ^10^Neurochemistry Laboratory, Department of Clinical Chemistry, Amsterdam Neuroscience, Amsterdam University Medical Center (UMC), Vrije Universiteit Amsterdam, Amsterdam, Netherlands; ^11^Department of Psychiatry and Neurochemistry, The Sahlgrenska Academy at the University of Gothenburg, Mölndal, Sweden; ^12^Clinical Neurochemistry Laboratory, Sahlgrenska University Hospital, Mölndal, Sweden; ^13^Department of Neurodegenerative Disease, University College London (UCL) Institute of Neurology, London, United Kingdom; ^14^UK Dementia Research Institute at University College London, London, United Kingdom; ^15^Hong Kong Center for Neurodegenerative Diseases, Hong Kong, China; ^16^Lewy Body Dementia Association, Lilburn, GA, United States; ^17^Department of Neurology and Center for Sleep Medicine, Mayo Clinic, Rochester, MN, United States; ^18^Department of Neurology, Massachusetts General Hospital, Boston, MA, United States; ^19^Department of Neurology, Mayo Clinic, Jacksonville, FL, United States; ^20^Department of Neurology, Georgetown University Medical Center, Washington DC, CA, United States; ^21^Department of Neurology and Neurological Sciences, Stanford University, Stanford, CA, United States; ^22^Department of Neurology, Johns Hopkins School of Medicine, Baltimore, MD, United States; ^23^Department of Neurology, Barrow Neurological Institute, Phoenix, AZ, United States; ^24^Barrow Neurological Institute and Muhammed Ali Parkinson Center, Phoenix, AZ, United States; ^25^Department of Neurology, University of Rochester, Rochester, NY, United States; ^26^Department of Neurology, University of Florida College of Medicine, Gainesville, FL, United States; ^27^Department of Neurology, Johns Hopkins University School of Medicine, Baltimore, MD, United States; ^28^Department of Neurology, University of North Carolina, Chapel Hill, NC, United States; ^29^Department of Neurology, University of Washington, Seattle, WA, United States; ^30^Parkinson's Disease Research, Education and Clinical Center, Michael J. Crescenz VA Medical Center, Philadelphia, PA, United States; ^31^Department of Neurology, Perelman School of Medicine, University of Pennsylvania, Philadelphia, PA, United States; ^32^Department of Neurological Sciences, Rush Medical College, Chicago, IL, United States; ^33^Department of Neurosciences, University of California, San Diego, San Diego, CA, United States; ^34^Department of Neurology, Comprehensive Center for Brain Health, University of Miami Miller School of Medicine, Miami, FL, United States; ^35^Shirley Ryan Abilitylab and Department of Physical Medicine and Rehabilitation and Neurology, Parkinson's Disease and Movement Disorders, Northwestern University Feinberg School of Medicine, Chicago, IL, United States; ^36^Department of Neurology, University of Colorado School of Medicine, Aurora, CO, United States; ^37^Columbia University Irving Medical Center, New York, NY, United States; ^38^Department of Neurology, Emory University School of Medicine, Atlanta, GA, United States; ^39^Lou Ruvo Center for Brain Health, Cleveland Clinic, Cleveland, OH, United States; ^40^Department of Neurology, University of Virginia, Charlottesville, VA, United States; ^41^Department of Neurology, Johns Hopkins University School of Medicine, Baltimore, MD, United States; ^42^Departments of Neurology and Ophthalmology, University of Colorado School of Medicine, Aurora, CO, United States; ^43^Department of Neurology, Ohio State University Wexner Medical Center, Columbus, OH, United States; ^44^Lou Ruvo Center for Brain Health, Cleveland Clinic Lerner College of Medicine, Las Vegas, NV, United States; ^45^Department of Neurology, Oregon Health and Science University, Portland, OR, United States; ^46^Department of Neurology, VA Portland Medical Center, Portland, OR, United States; ^47^Department of Neurology, University of Pennsylvania Health System, Philadelphia, PA, United States; ^48^Digital Neuropathology Laboratory, Philadelphia, PA, United States; ^49^Lewy Body Disease Research Center of Excellence, Philadelphia, PA, United States; ^50^Frontotemporal Degeneration Center, Philadelphia, PA, United States

**Keywords:** cerebrospinal fluid, alpha-synuclein, skin biopsy, seeded aggregation assays, tau, amyloid, Lewy body dementia, LBDA biomarker symposium

## Abstract

The Lewy Body Dementia Association (LBDA) held a virtual event, the LBDA Biofluid/Tissue Biomarker Symposium, on January 25, 2021, to present advances in biomarkers for Lewy body dementia (LBD), which includes dementia with Lewy bodies (DLBs) and Parkinson's disease dementia (PDD). The meeting featured eight internationally known scientists from Europe and the United States and attracted over 200 scientists and physicians from academic centers, the National Institutes of Health, and the pharmaceutical industry. Methods for confirming and quantifying the presence of Lewy body and Alzheimer's pathology and novel biomarkers were discussed.

## Introduction

The Lewy Body Dementia Association (LBDA) Research Centers of Excellence presented a virtual symposium on biomarkers for consideration in clinical trials on January 25, 2021. The goals were to identify biomarkers that may be useful as inclusion criteria and as outcome measures in clinical trials in Lewy body dementia (LBD) which is a major opportunity to improve clinical trials for these individuals (1). The discussion was confined to biomarkers in body fluids and tissues and did not extend to other modalities such as neuroimaging. Given that the majority of people with LBD harbor both Lewy body and Alzheimer's disease (AD) pathology (2–5), options for identifying and quantifying the presence of each of these types of pathology were reviewed. Novel biomarkers for related disease processes such as endolysosomal trafficking were also evaluated. Although the focus of the symposium was to assess candidate biomarkers for potential use in clinical trials, the role of biomarkers in disease pathophysiology was also considered.

## Biomarkers of Alpha-Synuclein Pathology

Two modalities for detecting pathologic alpha-synuclein were discussed: seeded aggregation assays (SAAs) and immunohistochemical detection of pathologic alpha-synuclein (Table 1). SAAs, also described as protein misfolding cyclic amplification (PMCA) or real-time quaking-induced conversion (RT-QuIC) assays, amplify small amounts of aggregated protein in body fluids or tissue homogenates in an iterative process of aggregation and partial disaggregation (6). Such assays have become a focus of alpha-synuclein biomarker research since measurement of total, phosphorylated, and oligomeric alpha-synuclein in cerebrospinal fluid (CSF) and serum has to date failed to demonstrate acceptable diagnostic value (14). SAAs are well developed as a clinically useful assay in prion disease, and several laboratories have now adapted the methods for detecting aggregated alpha-synuclein in biofluids (15–18). Dr. Lebovitz described published studies documenting that the specificity and sensitivity of the PMCA assay in CSF samples from clinically diagnosed individuals with Parkinson's disease (PD) is >90% (6, 17, 19). In dementia with Lewy bodies (DLBs), seeding activity is significantly increased over PD, so it is perhaps not surprising that test specificity and sensitivity are also >90% for individuals with DLB vs. controls and individuals with DLB vs. nonsynucleinopathies (16, 20, 21). Furthermore, certain diseases not typically classified as synucleinopathies have been shown to possess subtypes with incidental findings of Lewy body pathology. Previous studies utilizing SAA report that clinically diagnosed patients with AD were found to have aggregated synuclein in CSF in 5/14 cases (36%) (19). Until now, SAAs have been assessed only in clinically-diagnosed cases; however, evaluation of diagnostic value in pathologically confirmed individuals is underway.

**Table 1:** Fluid and tissue biomarkers of alpha-synuclein pathology.

**Biomarker**	**Utility for inclusion/exclusion criteria**	**Utility as an outcome measure**	**Procedure**	**Comments**
CSF, seeded aggregation assay for alpha-synuclein	**PD vs. Ctrl:** good SN, SP, AUC (0.89–0.95) (6) **PD/DLB/PAF**: good SN, SP (7) **RBD vs. Ctrl**: good SN, SP (8)	Changes over time not established. **PD**: MIF correlates with UPDRS score **RBD**: PD/DLB risk (8)	Lumbar puncture	Commercial test to be released in 2021
Skin biopsy, seeded aggregation assay for alpha-synuclein	**PD**: good SN/SP (9, 10) **PD/DLB/MSA vs. other**: good SN/SP (10)	Change over time not established **PD**: MIF correlates with UPDRS and MMSE (9)	Punch biopsy, frozen	Promising results, numbers of patients still modest, lower SN/SP for FFPE
Skin biopsy(-ies), IF synuclein & PGP 9.5	**PD:** good SN/SP when optimized (11) **PD/DLB/MSA/PAF vs. other:** good SN/SP, better for IF vs. CSF or skin RT-QuIC in one study (12)	One study shows 7% increase per year (unpublished)	1–3 punch biopsies, frozen or ZF	Promising results; needs multisite validation; commercial test offered since 2020
Skin biopsy-single biopsy (scalp), single-label IHC for alpha-synuclein	**PD:** good SP with optimized methods; SN limited (24%) especially in early PD. Better SN than colon (13)	Changes over time not established	Punch biopsy, FFPE	Not likely to be developed further
Submandibular gland biopsy	**PD**: good SP with optimized methods; limited SN (56%) especially in early PD; better SN than colon and skin (13)	Changes over time not established	Submandibular biopsy by ENT	Not likely to be developed further
Colon biopsy	**PD**: good SP with optimized methods; low SN (<15%) especially in early PD (13)	Changes over time not established	Colonoscopy with biopsy	Not likely to be developed further

*Designation of “good” means >90%. PD, Parkinson's disease; DLBs, dementia with Lewy bodies; PAF, pure autonomic failure; RBD, REM-behavior sleep disorder; UPDRS, Unified Parkinson's Disease Rating Score; MMSE, Mini Mental State Examination; SN, sensitivity; SP, specificity; AUC, area under the curve; MIF, maximum intensity fluorescence; FFPE, formalin-fixed paraffin embedded tissues; IF, immunofluorescence; ZF, Zamboni's fixative*.

In a cohort of 120 patients with a majority of AD clinical diagnoses, the PMCA assay, using antemortem CSF samples, correctly identified Lewy body pathology with a sensitivity of 61.5% and a specificity of 100%, when compared to autopsy findings (unpublished). Interestingly, when broken down by Lewy body distribution, people with AD who harbored Lewy bodies mainly in the amygdala at autopsy were detected only 13% (2/15) of the time with the PMCA assay, suggesting that this assay may not be suitable for certain subtypes of LBD (unpublished). Patients clinically diagnosed with PD or DLB and had autopsy-confirmed Lewy body pathology tested positive by the PMCA assay 4/4 (100%) and 6/8 (75%), respectively (unpublished). Quantitative aspects of the assays (e.g., time to amplification, maximal fluorescence) still require further testing. Data examining these metrics across individuals of advancing disease state (measured by postmortem alpha-synuclein), within individuals longitudinally, and with larger sample sizes are ongoing and are needed to demonstrate clinical utility.

Dr. Beach provided a history of efforts since 2007 to develop immunohistochemical detection of pathologic alpha-synuclein in peripheral tissues as a biomarker of PD. Initial studies in colon biopsies were limited by high false-positive rates and poor interrater reliability, but subsequent multicenter studies concluded that these problems could be addressed by the screening of multiple candidate methods and training of raters. Following such optimization, sensitivity and specificity of colon biopsy in autopsy tissue were excellent (100% accuracy for one method albeit in limited numbers of individuals) and could be useful diagnostically (13, 22, 23). However, the question of whether the colon was the best site for biopsy remained, due to an insufficient amount of submucosa obtained with current biopsy methods and invasiveness. Subsequent studies compared colon, submandibular gland, and skin biopsy. The Systemic Synuclein Sampling Study (“S4 Study”) employed consensus slide-reading by a panel of 5 specially trained neuropathologists, all blinded to diagnosis (13). The S4 Study found >90% specificity but disappointingly low sensitivity (56% in submandibular gland, 24% in skin, and very low sensitivity in colon). This may in part be attributable to the overrepresentation of early PD cases in the S4 Study: one-third of cases had a median disease duration of only 8 months, and the median disease duration for the entire PD group was 42 months (13). Sensitivity was greater in participants with more advanced PD, particularly in submandibular gland biopsies (76%). Dr. Beach concluded that improvement in the sensitivity of immunohistochemical methods was needed, or alternatively, SAAs such as RT-QuIC may soon supersede them. He hypothesized that whereas SAAs are already being done on CSF with very promising potential, peripheral tissue deposits may be a better model of brain tissue deposits and also perhaps a better measure of target engagement for monoclonal antibodies and other new therapeutic approaches.

Skin biopsy in the S4 Study consisted of two 3-mm skin punch biopsies obtained from the paravertebral posterior–inferior cervical area and mid-thigh; immunohistochemical labeling was performed with an alpha-synuclein antibody after protease pretreatment. Dr. Gibbons presented a distinct approach to skin biopsy involving three punch biopsies from the distal leg, distal thigh, and posterior cervical region in people with PD and DLB, using a double-label immunofluorescence method to detect and colocalize both alpha-synuclein and neuronal markers (PGP 9.5) in thick sections (11, 24, 25). His team found higher sensitivity than the S4 Study for a single biopsy (74%) and increased sensitivity when considering two (90%) or three (96%) biopsies (unpublished data presented at American Academy of Neurology Conference, 2020). They reported overall outstanding sensitivity and specificity for skin biopsy in this study and subsequent validation (accuracy 99.1%) as a laboratory-developed test, the “SYN-ONE” test (CND Life Sciences™), for discriminating peripheral synucleinopathies (PD, DLB, multiple system atrophy, pure autonomic failure) from controls (unpublished). In discussion, it was noted that Dr. Gibbons' unpublished data correlated with clinical scores including the Unified Parkinson's Disease Rating Scale (UPDRS), Orientation and Mobility Severity Rating Scale (OMSR), and DLB Cognitive Scale (unpublished). Biopsy acquisition was performed, using the SYN-ONE test, as part of a multicenter center study supported by the NIH Office of Rare Diseases (Autonomic Disorders Consortium). An additional multisite blinded study funded by NIH is currently underway to evaluate how this approach fares in the environment of multiple academic and private practice sites with a core reference laboratory-developed test. The skin biopsies prepared in this manner can also be quantified, and the first quantitative studies show significant autonomic and sensory nerve fiber density differences between groups, individuals with DLB having the most severe autonomic and sensory neuropathies, followed by idiopathic PD, and finally multiple system atrophy (MSA), without evidence of peripheral nerve degeneration. Dr. Gibbon's group also recently reported that cutaneous phospho-alpha-synuclein is moderately correlated (*r* = 0.6) with both sympathetic and total autonomic impairment in individuals with isolated REM sleep behavior disorder (iRBD) and is more common in iRBD with hyposmia (26).

The final speaker in this session, Dr. Kanthasamy, linked the SAA and tissue biopsy approaches by presenting results from skin biopsy samples that were analyzed not with immunohistochemistry but instead homogenized and processed with RT-QuIC. After establishing the method with autopsied brain tissue and submandibular gland (15, 27), his group compared skin samples from individuals with PD and controls and found that the SAAs performed on skin homogenates yielded a specificity of 96% and sensitivity of 96% (9). The maximal fluorescence metric from the seeding assay also correlated with disease severity (UPDRS *p* < 0.0001; Mini-Mental Status Examination (MMSE) *p* = 0.0035), suggesting that quantitative aspects of this assay might be useful as a marker of disease state and potentially as an outcome measure in clinical trials targeting alpha-synuclein. An advantage of seeding assays over immunohistochemistry is that they do not require extended review by specially trained neuropathologists.

## Biomarkers for Alzheimer's Disease Pathology

Dr. Lemstra provided a summary of CSF biomarkers for AD pathology in LBD (Table 2). Studies in neuropathologically-confirmed cases have shown that mixed pathology can be detected antemortem with CSF biomarkers using similar cutoffs employed for AD. Larger *in vivo* cohorts including the European DLB Consortium (E-DLB), Mayo Clinic Cohort, and Amsterdam Dementia Cohort have shown that AD biomarkers, either CSF (most commonly CSF tau/Aβ42 ratio) or PET markers, are increased in DLB over PD and PDD and correlate with DLB dementia, progression, and survival. Evidence suggests that these Alzheimer markers likely reflect concomitant Alzheimer pathological process along with the Lewy body disease. Specifically, AD biomarker in DLB (DLB-AD) is defined by the presence of AD biomarkers unlike DLB-pure (DLB-p) and is a common subtype (at least 50%) of DLB characterized by more rapid clinical deterioration and mortality (33). DLB-AD is associated with increased age, female sex, increased *APOE* ε4 genotype, decreased memory, increased delusions and hallucinations, less REM-behavior sleep disorder and parkinsonism, worse language performance, faster progression, increased temporal thinning and tau pathology, and greater risk of institutionalization and mortality (2–4, 33–37). These findings are corroborated by postmortem clinicopathologic studies which show that AD pathologic features (neuritic plaques and also tangles) in cases clinically defined as “probable DLB” are associated with an atypical “Alzheimerized” clinical presentation (e.g., worse performed on orientation and memory testing) (57, 58). Dr. Lemstra also presented studies that show CSF AD biomarkers do not appear to influence positivity rates of DaTscan (59) or electroencephalography (60) but emphasized that more studies are needed. Different amyloid-beta species were also discussed as possible biomarkers for discriminating DLB from AD. Unlike AD, in which Aβ42 is more selectively decreased, multiple studies have shown that DLB exhibits a broader decrease of multiple amyloid-beta species (Aβ38, Aβ40, Aβ42). Furthermore, these studies show that some species of amyloid-beta (Aβ38, Aβ40) decrease independently of AD biomarkers (CSF tau/Aβ42) and *APOE* genotype, and some species (Aβ38) correlate with disease duration. Ratios (Aβ42/Aβ38) can discriminate clinical DLB from AD with moderate accuracy (sensitivity 78%, specificity 67%) (28, 29). Limited data suggest a negative association between symptomatic treatment with acetylcholinesterase inhibitors in DLB and longitudinal changes in AD biomarkers (61). It was proposed that DLB-AD could be considered for recruitment into clinical trials for amyloid-modifying therapy in the research setting, but future study is needed to clarify the relationship between amyloid deposition and clinical symptoms in AD and DLB-AD.

**Table 2:** Fluid and tissue biomarkers of AD pathology.

**Biomarker**	**Utility for inclusion/exclusion criteria**	**Utility as an outcome measure**	**Procedure**	**Comments**
CSF Aβ, tau, pTau	**DLB-AD** **>** **DLB-p**: tTau/Aβ_1−42_ good sensitivity and specificity (2) **DLB vs. AD**: Aβ42/Aβ38 modest sensitivity and specificity (AUC 0.76) (28, 29) **AD vs. MCI/Ctrl**: core biomarkers, improve detection of early stages (30, 31)	**PD:** Progression (32) **DLB**: Changes over time are not established **DLB-AD vs. DLB-p** tTau/Aβ_1−42:_ worse progression (2–4, 33–37) **AD**: Prodromal / baseline Aβ and tau related to later atrophy, amyloid, decline (38–41)	Lumbar puncture	**AD**: soon universal Aβ cutoffs. Timecourse data marks AD subtypes (39, 42); indicates drug-target engagement (43, 44)
CSF NfL	**DLB**: Sensitive and early marker, nonspecific (45). Elevations in DLB > Ctrl (AUC 0.94), proDLB > Ctrl (AUC 0.87), DLB > proDLB (AUC 0.6), DLB-AD > DLB-p (lower AUC vs. tau/Aβ, not shown) (46) **MSA, PSP, CBS** **>** **other parkinsonian** dz (45, 47) **AD**: sensitive, nonspecific (31)	**DLB**: Changes over time are not established **AD**: Correlates with degeneration (48); increase with dementia, decline, atrophy (46, 49).	Lumbar puncture	
Plasma Aβ42/40	**DLB**: Aβ42 unchanged in small study (50); ratio not studied **AD**: Small effect but good AUC (~0.85), complements covariates (*APOE* ε4, age)	Changes over time are not established	Blood draw	AUC better for mass spec vs. IA; however stability unproven
Plasma tTau/pTau	**DLB**: Nonspecific for DLB, small study, ratio untested (50); predicts abnormal tau-PET and CSF Aβ42/Aβ40 (51) **AD vs. Ctrl, NDD**: pTau181 and pTau217 accurate (AUCs) (0.87 – 0.98); DLB part of NDD controls (52)	**DLB**: Changes over time are not established **AD**: pTau181 and pTau217 predict and correlate with ongoing progression (52–54); more amyloid-specific than plasma NfL	Blood draw	Head-to-head comparison needed
Plasma NfL	Nonspecific marker of damage (54, 55) **DLB and proDLB vs. Ctrl:** Elevated but unclear test performance (preprint) (56)	**DLB**: Predicts cognitive progression (preprint) (56); changes over time not established **AD**: Progression independent of tau but not AD-specific (54)	Blood draw	

*Designation of “good” means >90%. AD, Alzheimer's disease; PD, Parkinson's disease; DLBs, dementia with Lewy bodies; DLB-AD, dementia with Lewy bodies and AD biomarkers; DLB-p, “DLB-pure”/dementia with Lewy bodies and lacking AD biomarkers; proDLB, prodromal DLB; MSA, multiple system atrophy; PSP, progressive supranuclear palsy; CBS, corticobasal syndrome; tTau, total tau; pTau, phosphorylated tau; NDD, neurodegenerative diseases/dementias; NfL, neurofilament light chain; AUC, area under the curve; Ctrl, controls; dz, diseases; IA, immunoassay*.

The final speaker, Dr. Shaw, summarized progress in blood biomarkers for AD, mainly focusing on targets whose studies are well underway (Aβ42/40, pTau181 and 217, neurofilament light chain [NfL]), with a brief mention of earlier-stage targets (alpha-synuclein, TDP-43, GFAP, NPTX2). Plasma Aβ42/40 as a biomarker shows small but reproducible absolute differences in amyloid-positive (by PET) vs. amyloid-negative patients and shows early promise as a screening test alongside covariates, *APOE* ε4 genotype, and age. In general, the area under the receiver operating characteristic curve (AUC) has been better in mass spectrometry studies (AUC 0.82–0.89) (62, 63) than immunoassays (AUC 0.65–0.77, up to 0.80 when Aβ42 and Aβ40 were used in a logistic regression model instead of the Aβ42/Aβ40 ratio) (64). A large head-to-head round robin study [Foundation for the National Institutes of Health Biomarkers Consortium (FNIH BC)/Alzheimer's Disease Neuroimaging Initiative (ADNI)] nearing completion will compare 3 mass spectrometry and 3 immunoassays for plasma Aβ42/40 ratio concordance with amyloid-PET. pTau181 and pTau217, tested by immunoassay or by mass spectrometry, can reliably detect tau pathology and levels correlate with amyloid PET, cerebral atrophy, and cognitive decline (53, 65). Some reports suggest that pTau217 has somewhat superior sensitivity and specificity vs. pTau181 for discriminating AD from other disorders and healthy controls (66), but more head-to-head studies are needed to definitively address this question. Preanalytical, analytical, and clinical replication studies are underway in international groups, and there are multiple companies developing diagnostic tests for these targets. Dr. Shaw also highlighted the importance of detailed preanalytical studies to test variables such as delayed centrifugation at room temperature and freeze-thaw cycles that can affect these measurements. The Alzheimer's Association Global Biomarker Standardization Consortium recently reported that these findings at the 2021 Alzheimer's Association International Conference, underscoring the need to resolve interlaboratory differences before widespread clinical applications, are implemented. NfL was also briefly mentioned as a third well-studied biomarker of nonspecific neurodegeneration that needs larger head-to-head confirmatory studies. During the discussion, other promising pTau targets were mentioned including pTau231 and diphosphorylated peptides. Low DLB enrollment in these studies was noted, and ongoing consortia were highlighted that are measuring pTau181, pTau231 and Aβ42/40, NfL, and glial fibrillary acidic protein (GFAP). Newer CSF and plasma biomarkers for AD, Lewy body, and non-AD neurodegenerative disorders are also reviewed elsewhere (67).

## Biomarkers of Other Disease Mechanisms

Two modalities for the discovery of novel CSF biomarkers were discussed: mass spectrometry and antibody array proteomics (Table 3). Mass spectrometry is an unbiased approach with longstanding precedent in laboratory testing and biomarker research (e.g., plasma AD markers discussed above), whereas newer antibody arrays, such as O-link® Proximity Extension Assay (PEA) discussed below, offer more targeted, higher throughput, and potentially more sensitive multiplexed immunoassays. Interestingly, these technologies have been shown to cover different fractions of the proteome, leading to partly complementary results (70).

**Table 3:** Fluid and tissue biomarkers for other disease mechanisms.

**Biomarker**	**Utility for inclusion/exclusion criteria**	**Utility as an outcome measure**	**Procedure**	**Comments**
CSF autophagy markers	**DLB**: Not established **AD**: Increase **PD:** Decrease	**DLB:** Changes over time not established	Lumbar puncture	
Novel CSF biomarkers NPTX2, VGF	**DLB vs. Ctrl:** • NPTX2 (AUC 0.81) • VGF (AUC 0.81) • NPTX2+VGF+age+ αSyn (AUC 0.94) **AD vs. Ctrl/MCI:** NPTX2 (68)	**DLB:** Low VGF associated with cognitive decline at time of presentation, high VGF associated with future cognitive decline (69); changes over time are not established **AD:** NPTX2 change correlates with cognitive decline (68)	Lumbar puncture	VGF correlated with CSF tau and αSyn

Dr. Zetterberg presented CSF mass spectrometry data of a panel of proteins involved in endolysosomal and autophagosome processing. These fundamental intracellular sorting organelle systems have been implicated by genetic and histologic studies in DLB [reviewed in Arotcarena et al. (71)] but have rarely been studied in CSF. Dr. Zetterberg presented data from the measurement of these proteins quantitatively in AD and PD and achieved simultaneous measurement of 18 related proteins in approximately 0.2 ml of CSF. The team's results show a pattern of increase in AD vs. controls for many lysosomal markers (e.g., cathepsin B, LAMP2) and endocytosis (AP2) and ubiquitin whereas there was a decrease in PD for the same group of proteins. The causes of increased protein release in AD and decreased release in PD are unknown but results argue against nonspecific neurodegeneration since results for AD and PD are in the opposite direction. Discussants noted similar decreases across multiple proteins (synuclein, tau, Aβ) in the Parkinson's Progression Markers Initiative (PPMI) cohort and one speculated unifying explanation was retromer dysfunction (72). Studies in patients with DLB have not yet been performed using this approach.

The second speaker, Dr. Teunissen, first discussed biomarker discovery in DLB using mass spectrometry and ELISA validation. Six potential targets were identified in DLB vs. control including downregulation of neurosecretory protein VGF, neuronal pentraxin-2 (NPTX2), neuroendocrine convertase 2 (PCSK2), neuronal pentraxin receptor (NPTXR), upregulation of ubiquitin carboxyl-terminal hydrolase (USP14), and proteasome subunit beta type-7 (PSMB7) (73). The extent of downregulation of NPTX2 and VGF in DLB was greater than but overlapped with the extent of downregulation in AD and PD. It is unclear whether DLB patients with lower NPTX2 have more amyloid pathology, a potentially important distinction given that AD studies (e.g., ADNI) also find NPTX2 dysregulation (68). In the second half of his presentation, Dr. Teunissen presented early results of a broad search for distinguishing a DLB subgroup with AD CSF biomarkers (DLB-AD) using multiplexed immunoassay arrays, Olink®, discussed briefly above. Interestingly, Dr. Teunissen's early data disagree with the hypothesis that biomarkers for DLB-AD represent a simple combination of biomarkers for Lewy body disease plus AD. The team found that DLB-AD had unique features compared to DLB-p and AD, including lower CSF protein levels and specific protein differences. In contrast, few differences in proteins were observed between DLB-p and AD in these data. The identity of these proteins enriched in DLB-AD included cell adhesion, cytokine–cytokine interactions, axon guidance, and neurogenesis. Preliminary validation studies with a more quantitative immunoassay, the Ella® system, show replication of Olink® hits and further discriminatory power of candidate proteins in addition to pTau and tTau in distinguishing DLB from AD.

## Conclusion and Future Directions

### Alpha-Synuclein Biomarkers

There was agreement by the presenters regarding the urgent need for sensitive and specific *in vivo* biomarkers to detect alpha-synuclein pathology, which has the potential to improve therapeutic targeting and also to inform disease pathogenesis. Panel discussants provided insights into the logistics and needs for further validation of emerging peripheral tissue biopsies and SAAs for these approaches to reach utility in therapeutic trials and eventually clinical use. Key gaps include the need for further autopsy validation, longitudinal analyses, and standardization of assay methods. Indeed, there are animal and cell-model data which suggest that alpha-synuclein pathology can spread throughout the nervous system (74, 75), but it remains unclear whether and how this occurs in humans. The Braak staging system for PD suggests that pathology begins in the peripheral autonomic nervous system or olfactory bulb and then migrates proximally to the amygdala and brainstem and in the more severe cases, to the cerebral cortex (76). This model is supported by some human studies, such as those of multiple groups finding high levels of alpha-synuclein pathology in skin biopsies in early PD and REM-sleep behavior disorder (RBD) without other clinical evidence of synucleinopathy (12, 25, 26, 77–79). However, the model is not supported by other large autopsy-based studies that find no evidence of peripheral-first synucleinopathy (e.g., stomach vs. brain) and that peripheral synucleinopathy is more common and severe in later stages (80, 81). Overall, these observations combined with the fact that limbic Lewy body pathology may occur prior to brainstem pathology in the course of DLB, indicate that the initiation and propagation of Lewy body pathology is varied among the synucleinopathies (82) and may vary by predictors such as genotype, and that future study is needed to resolve these findings.

### AD Biomarkers in LBD

In contrast to biomarkers of alpha-synuclein, sensitive AD biomarkers are established and provide a link between clinical and pathologic aspects of DLB with and without AD pathology. Panelists agreed with the potential importance of stratifying therapeutic trial inclusions and/or outcomes based on AD biomarker profiles in LBD due to the strong association of these biomarkers with clinical outcomes, exemplified by the prospective data in the European DLB (E-DLB) consortium. The frequency of AD pathology in DLB (DLB-AD) varies between 25 and 89% depending on biomarker cutoffs and diagnostic criteria (2–5). Distinct lines of evidence have shown that DLB-AD may represent a biological interaction of these mixed amyloid-beta, tau, and alpha-synuclein pathologies. In clinically defined DLB, unpublished data suggest that DLB-AD appears to exhibit a distinct CSF immunophenotypic pattern, raising the importance of exploratory biomarker discovery work to further refine biological subgroups in DLB. Moreover, in autopsy-defined DLB, there is lower overall tau compared to AD and higher temporal lobe enrichment of tau that is associated with both cortical thinning and cognitive impairment (37, 83). Although the longitudinal assessment of AD biomarker progression in DLB is understudied, there are conflicting results in PD (32, 84) that appear to be explained by variable disease stages and methods of measurement in individual studies, but also intrinsic biological variability between patients. Thus, future prospective longitudinal DLB-specific studies with autopsy-confirmation are needed to quantitatively compare the time course of amyloid and tau biomarkers and to consider potential DLB-specific cutoffs. By enabling the study of homogenous patient populations with similar underlying biology, these efforts will increase the capacity to assess treatment outcomes in DLB-focused clinical trials.

Overall, rapid progress has been made in the development of fluid and tissue-based biomarkers for Lewy body dementia and they show promise as useful tools. Further external validation and translational research are needed specifically in individuals with Lewy body dementia to accurately determine biomarker test characteristics and overall determine how these individuals may benefit from such a biomarker test.

## Author Contributions

All authors listed have made a substantial, direct, and intellectual contribution to the work and approved it for publication.

## Funding

This study is supported by the Lewy Body Dementia Association.

## Conflict of Interest

TB has conducted consultation for a peripheral synuclein assay and speakers honorarium from Roche Diagnostics. RL is employed full time by Amprion, serves on Amprion's Board, and is a shareholder. CT research is supported by the European Commission (Marie Curie International Training Network, grant agreement No 860197 (MIRIADE), and JPND), Health Holland, the Dutch Research Council (ZonMW), Alzheimer Drug Discovery Foundation, the Selfridges Group Foundation, Alzheimer Netherlands, Alzheimer Association. CT is the recipient of ABOARD, which is a public-private partnership receiving funding from ZonMW (#73305095007) and Health Holland, Topsector Life Sciences & Health (PPP allowance; #LSHM20106). More than 30 partners participate in ABOARD. ABOARD also receives funding from Edwin Bouw Fonds and Gieskes Strijbisfonds. IV is appointed on a research grant by Alzheimer Nederland (NL 17004). CT has a collaboration contract with ADx Neurosciences, Quanterix, and Eli Lilly, who performed contract research or received grants from AC Immune, Axon Neurosciences, Biogen, Brainstorm Therapeutics, Celgene, EIP Pharma, Eisai, PeopleBio, Roche, Toyama, Vivoryon. CT serves on editorial boards of Medidact Neurologie/Springer, Alzheimer Research and Therapy, Neurology: Neuroimmunology and Neuroinflammation, and is editor of a Neuromethods book by Springer. HZ has served at scientific advisory boards and/or as a consultant for Abbvie, Alector, Eisai, Denali, Roche Diagnostics, Wave, Samumed, Siemens Healthineers, Pinteon Therapeutics, Nervgen, AZTherapies, CogRx, and Red Abbey Labs has given lectures in symposia sponsored by Cellectricon, Fujirebio, Alzecure and Biogen, and is a co-founder of Brain Biomarker Solutions in Gothenburg AB (BBS), which is a part of the GU Ventures Incubator Program (all outside submitted work). CMo is an inventor of several US and International Georgetown University patents to use tyrosine kinase inhibitors (TKis) for the treatment of neurodegenerative diseases. CMo is a co-founder and shareholder and receives consulting fees from KeifeRx LLC. CMo receives consulting fees from Neumentum LLC, SUn Pharmaceuticals Research Industry, and SkyBIo. CMo received NIH NIA funding, Alzheimer's Association, and Sun Pharmaceuticals Research Industry Funding to study TKis in neurodegeneration, including LBD. KP has received consulting fees from Curasen and is funded by grants from the NIH, Michael J Fox Foundation for Parkinson's Research, LBDA, and Alzheimer's Drug Discovery Foundation, and has received funding from Sanofi US Services, Inc. to perform clinical trials. MS declares ownership interest (Stock or stock options): Brain Health Inc, NeuroTau, Optimal Cognitive Health Company, uMethod Health, Versanum, Athira, Cognoptix and consulting work for Alzheon, Biogen, Cortexyme, Roche Genentech, Stage 2 Innovations/Renew Research, Acadia, T3D, Eisai, KeifeRx. MS also declares royalties: HarperCollins, Humanix and speakers bureau: Health and Wellness Partners. LR receives grant funding from the NINDS, Michael J. Fox Foundation, Parkinson's Foundation, and National Ataxia Foundation. She has also served on advisory boards for Uniqure and other pharmaceutical companies through the Parkinson's Study Group. MJA receives research support from the NIA (R01AG068128, P30AG047266), the Florida Department of Health (Grant 20A08), and as the local PI of a Lewy Body Dementia Association Research Center of Excellence. MJA serves on the data safety monitoring boards (DSMBs) for ACTC/ATRI and ADCS. MJA receives royalties from the publication of the book Parkinson's Disease: Improving Patient Care. MJA serves on the level of evidence editorial board for Neurology and related publications (uncompensated). JGG declares grants/research—Acadia, Michael J. Fox Foundation, Parkinson's Foundation, Consultant—Worldwide Med, Honoraria—American Academy of Neurology, Davis Phinney Foundation, International Parkinson and Movement Disorders Society, Medscape, Parkinson's Foundation, Other: Lewy Body Dementia Association Research Center of Excellence. ZM received institutional grant support from NIH, MJFF, LBDA, Parkinson's Foundation, Eli Lilly, NeuroDerm, Cerevel Therapeutics and personal consulting honoraria from Global Kinetics Corporation, GB Sciences, ACADIA, PSG, Elsevier, Kyowa Kirin, AbbVie, and Supernus. IL's research is supported by the National Institutes of Health Grants: 2R01AG038791-06A, U01NS100610, U01NS80818, R25NS098999; U19 AG063911-1 and 1R21NS114764-01A1; the Michael J Fox Foundation, Parkinson Foundation, Lewy Body Association, CurePSP, Roche, Abbvie, Biogen, Centogene. EIP-Pharma, Biohaven Pharmaceuticals, Novartis, Brain Neurotherapy Bio, and United Biopharma SRL—UCB. IL was a member of the Scientific Advisory Board of Lundbeck and is a Scientific Advisor for Amydis and Rossy Center for the Progressive Supranuclear Palsy University of Toronto. IL receives her salary from the University of California San Diego and as Chief Editor of Frontiers in Neurology. JL receives grant funding from the NIH/NIA/NINDS (P30AG072959/P30AG062428/U01NS100610), LBDA, GE Healthcare and serves as a consultant for Eisai Pharmaceuticals. RW serves on the data safety monitoring boards for Alexion, Bukwang, and Sarepta Pharmaceuticals and the Scientific Platform Steering Committee for Adams Pharmaceuticals. RW serves as a consultant for Lundbeck, Acadia, Teva, Abbvie, Alexion, Adamas, Prime, Bukwang, Sarepta, Syneos, Techspert, Guidepoint and also for R01R01NS117547 with Principal Investigator, Virendra Mishra. AL has received research support from the Dutch Research Council (ZonMW), Alzheimer Netherlands, Dioraphte Foundation, AL has given lectures in symposia sponsored by GE. CG is a scientific consultant and has stock in CND Life Sciences. SG has served on Advisory Boards of Jannsen, Acadia, and Sanofi, has received consulting fees from EIP Pharma, and has received funding from the NIH, the DOD CDMRP, the Michael J. Fox Foundation, the FFFPRI, and the Lewy Body Dementia Association. BB has served as an investigator for clinical trials sponsored by Alector, EIP Pharma, and Biogen. He serves on the Scientific Advisory Board of the Tau Consortium. He receives institutional research support from the NIH, the Lewy Body Dementia Association Research Centers of Excellence Program, the Mayo Clinic Dorothy and Harry T. Mangurian Jr. Lewy Body Dementia Program, the Little Family Foundation, and the Ted Turner and Family Foundation. AP serves on the Scientific Advisory Board of MedRhythms, Inc. and receives research support from the Lewy Body Dementia Association (as Co-Director of the Johns Hopkins LBDA Research Center of Excellence) and NIH/NINDS/NIA (U01NS102035; K23AG059891). DG receives research support from NIA (AG062429), the State of California, and the Michael J. Fox Foundation, which is a paid consultant for Biogen, Roche, Esai, Cognition Therapeutics, Fujirebio, Amprion, Generian, and Editor of Alzheimer's Research & Therapy. DH is supported by The Michael J. Fox Foundation (MJFF-010556), NIH-NINDS 1K23NS105944-01A1, the American Parkinson's Disease Association Center for Advanced Research (Emory University), and the Lewy Body Dementia Association Research Center of Excellence (Emory University). JF is on the editorial board of the American Academy of Neurology's Brain and Life magazine (uncompensated), Lewy Body Dementia Association Scientific Affairs Committee (uncompensated), and has received honoraria from Parkinson's Foundation. JF receives grant funding from the NIH/NIA/NINDS (K23NS097615, 5P30AG064200-02), CurePSP, and Parkinson's Foundation. KD-R receives research funding from the NIH (P30 AG066509) and LBDA Research Center of Excellence; has served on a Biogen advisory board; receives speakers honoraria from MedBridge. LS receives research support from NIH/NIA U19 AG024904, ADNI3 grant; NIH/NIA P30 AG010124, UPENN ADCC grant; and the Michael J. Fox Foundation for Parkinson's Research. He is a consultant for Biogen and Roche Diagnostics and is on the speaker's bureaus for Biogen and Fujirebio. DI receives research funding from NIH (R01-NS109260, P01-AG066597, U19-AG062418, U01-NS100610), Penn Institute on Aging and is the co-PI of the Penn LBDA Research Centers of Excellence. He is a member of the scientific advisory board of Denali Therapeutics. LH is a paid consultant for Biogen, Cortexyme, Eisai, Medscape, and Prevail. LH receives grant support from NIA, NINDS, LBDA, Acumen, Alector, Avanir, Eisai, Genentech/Roche, Janssen/Johnson & Johnson, NovoNordisk, Transposon, UCB, and Vaccinex. JD receives research funding from NIH, the Department of Veterans Affairs, the Michael J. Fox Foundation and Innervace, Inc. He is also the co-PI of the Penn LBDA Research Center of Excellence. IL is the Editor in Chief of the journal but she was not involved in the process of referee selection, peer review or the acceptance decision. The remaining authors declare that the research was conducted in the absence of any commercial or financial relationships that could be construed as a potential conflict of interest.

## Publisher's Note

All claims expressed in this article are solely those of the authors and do not necessarily represent those of their affiliated organizations, or those of the publisher, the editors and the reviewers. Any product that may be evaluated in this article, or claim that may be made by its manufacturer, is not guaranteed or endorsed by the publisher.
